# Role of multidrug resistance protein 2 (MRP2) in chemoresistance and clinical outcome in oesophageal squamous cell carcinoma

**DOI:** 10.1038/sj.bjc.6606071

**Published:** 2011-01-04

**Authors:** M Yamasaki, T Makino, T Masuzawa, Y Kurokawa, H Miyata, S Takiguchi, K Nakajima, Y Fujiwara, N Matsuura, M Mori, Y Doki

**Affiliations:** 1 Department of Gastroenterological Surgery, Graduate School of Medicine, Osaka University, 2-2-E2, Yamada-oka, Suita, Osaka 565-0871, Japan; 2 Department of Molecular Pathology, School of Allied Health Science, Faculty of Medicine, Osaka University, Osaka, Japan

**Keywords:** multidrug resistance protein 2, MRP2 expression, prognosis, oesophageal squamous cell carcinoma, chemoresistance, neo-adjuvant chemotherapy

## Abstract

**Background:**

Although multidrug resistance protein 2 (MRP2) confers chemoresistance in some cancer types, its implication on oesophageal squamous cell carcinoma (ESCC) remains unclear.

**Methods:**

We evaluated MRP2 expression by immunohistochemistry and RT–PCR using 81 resected specimens from ESCC patients who did or did not receive neo-adjuvant chemotherapy (NACT), including 5-fluorouracil, doxorubicin, and cisplatin (CDDP). Correlation between MRP2 expression and response to chemotherapy was also examined in 42 pre-therapeutic biopsy samples and eight ESCC cell lines.

**Results:**

MRP2-positive immunostaining was more frequently observed in ESCCs with NACT than in those without NACT (27.3 *vs* 5.4%). The MRP2-positive patients showed poorer prognosis than MRP2-negative patients (5-year survival rate, 25.6 *vs* 55.7%). Concordantly, ESCC with NACT showed 2.1-fold higher mRNA expression of MRP2 than those without NACT (*P*=0.0350). In pre-therapeutic biopsy samples of patients with NACT, non-responders showed 2.9-fold higher mRNA expression of MRP2 than responders (*P*=0.0035). Among the panel of ESCC cell lines, TE14 showed the highest MRP2 mRNA expression along with the strongest resistance to CDDP. Inhibition of MRP2 expression by small-interfering RNA reduced chemoresistance to CDDP.

**Conclusion:**

Our data suggested that MRP2 is one of molecules, which regulate the sensitivity to chemotherapy including CDDP in advanced ESCC patients.

Oesophageal squamous cell carcinoma (ESCC) is the major histological form of oesophageal cancer in East Asian countries. It is one of the most lethal malignancies of the digestive tract and in most cases the initial diagnosis is established only once the malignancy is in the advanced stage (Shimada *et al*, 2003). Multimodal therapies are therefore necessary to prolong the survival of ESCC patients. Chemotherapy has become the standard first-line therapy for advanced ESCC patients, especially neo-adjuvant chemotherapy (NACT) (Tamoto *et al*, 2004). However, the initial response rate for NACT remains at 35–66% (Ajani *et al*, 1992; Iizuka *et al*, 1992; Hilgenberg *et al*, 1988; Ilson *et al*, 1998, 1999; Millar *et al*, 2005) and non-responders risk serious adverse effects without achieving any survival benefit.

The effectiveness of chemotherapy is often limited by drug-resistance factors in the tumours themselves. In fact, some tumours are intrinsically resistant to many kinds of chemotherapeutic agents, whereas other tumours, initially sensitive, often recur or become resistant not only to the initial agents used but also to those used subsequently. These two types of chemoresistance, intrinsic and acquired, are clinically serious problems in many types of cancer including ESCC; however, the molecular mechanisms underlying this resistance are not fully understood. More investigation into the mechanisms of chemoresistance in ESCC is needed with the goal of identifying novel predictive markers that can accurately identify non-responders before the administration of chemotherapy, thus enabling personalised therapies in ESCC patients.

Several members of the ATP-binding cassette (ABC) transporter superfamily have an important role in drug resistance in tumour cell models as well as in the clinic (Lage, 2003). These transporters mediate the ATP-dependent cellular efflux of chemotherapeutic drugs. Of the 48 human ABC transporters, multidrug resistance protein 2 (MRP2; also designated as ABCC2 or cMOAT) is expressed in the hepatocyte canalicular membrane (Kool *et al*, 1997), in which it functions as the major exporter of organic anions from the liver into the bile (Wada *et al*, 1998). Multidrug resistance protein 2 is also expressed in the kidney, gall bladder, small intestine, colon, and lung (Surowiak *et al*, 2006). Interestingly, several cisplatin (CDDP)-resistant human cancer cell lines overexpress MRP2, including ovarian cancer, hepatocellular carcinoma, bladder cancer, and colon cancer (Taniguchi *et al*, 1996; Kool *et al*, 1997; Liedert *et al*, 2003; Materna *et al*, 2005). *In vitro* data also implicated MRP2 in multidrug resistance (MDR) mechanisms during chemotherapy in some cancer cell lines (Koike *et al*, 1997; Materna *et al*, 2006; Ma *et al*, 2009). However, few studies have investigated MRP2 expression in ESCC (Gan *et al*, 2010; Tanaka *et al*, 2010), and thus the relationship between MRP2 expression and chemoresistance in ESCC remains unclear. The present study examined the clinical significance of MRP2 expression and its role in intrinsic and acquired resistance to chemotherapy in ESCC patients.

## Patients and methods

### Patients and treatments

The present study examined samples from 81 patients with histopathologically confirmed primary thoracic oesophageal cancer who underwent surgical resection at our hospital from 1988 to 2007. Table 1 details the patient characteristics. The cohort comprised 9 female and 72 male patients, aged from 42 to 80 years (median 62 years). Sub-total oesophagectomy by right thoracotomy with two or three-field lymphadenectomy was performed in all patients. Curative resection (R0) was achieved in 75 patients (92.6%), whereas the remaining 6 (7.4%) patients underwent a non-curative resection (R1, 2). None of the patients died of post-operative complications. A total of 44 patients (54.3%) with lymph node metastasis at initial diagnosis received NACT comprising two courses of 5-fluorouracil (5-FU), CDDP, and doxorubicin (DXR) (Akita *et al*, 2006; Yano *et al*, 2006; Matsuyama *et al*, 2007; Makino *et al*, 2008, 2010). Only a few patients who showed multiple metastatic lymph nodes in the surgical specimen received a regimen of docetaxel or CDDP plus 5-FU after operation (Ando *et al*, 2003).

After surgery, the patients were surveyed every 3 months by physical examination and measurement of serum tumour markers, every 6 months by CT scan and abdominal ultrasonography, and every year by endoscopy until tumour recurrence was evident. Patients with tumour recurrence received chemotherapy or chemoradiotherapy as long as their systemic condition permitted. The mean overall survival (OS) was 31.6 months and mean disease-free survival was 28.3 months. The mean follow-up period after surgery was 42.9 months.

### Immunohistochemical analysis

MRP2 protein accumulation was examined by immunohistochemical (IHC) staining of formalin-fixed and paraffin-embedded ESCC tissue sections (Makino *et al*, 2009). Briefly, after de-paraffinization in xylenes and dehydration through graded ethanol solutions; endogenous peroxidase activity was blocked by incubation with 3% hydrogen peroxide for 20 min. The tissue sections were then heated at 95°C for 40 min in citrate buffer (0.05 mol l^−1^, pH 6.0) for antigen retrieval. The sections were then incubated with mouse monoclonal antibody to MRP2 (Clone: M_2_III-6, ALEXIS Biochemicals, dilution 1 : 10) for 2 h at room temperature, and antibody binding was visualised using the labeled-streptavidin biotin method. Negative controls for the IHC included omission of the primary antibody. Normal human liver tissue was used as a positive control. MRP2 staining for each ESCC sample was judged ‘positive’ when more than 10% of the cancer cells in the section were immunoreactive to MRP2, and ‘negative’ when 10% or less of the cells were positive. All slides were assessed by two observers, independently and then in conference; both were blinded to the clinico-pathological parameters.

### Quantitative RT–PCR analysis

Total RNA was extracted from fresh frozen resected tumours or endoscopic biopsy samples from ESCCs patients, and from cancer cell lines using TRIzol Reagent (Invitrogen, Carlsbad, CA, USA). Complementary DNA (cDNA) was generated from 1 *μ*g RNA in a final volume of 20 *μ*l containing oligo-(dT)-15 primer and avian myeloblastosis virus transcriptase, using the Reverse Transcription System (Promega, Madison, WI, USA). Analysis by PCR was performed using a LightCycler, real-time monitoring thermal cycler. Reaction mixture for PCR was prepared containing 2 *μ*l of cDNA template, 3 mmol l^−1^ MgCl_2_, and 250 nmol l^−1^ of primer pairs, using LightCycler FastStart DNA Master SYBR Green I (Roche Diagnostics, Mannheim, Germany). The amount of each transcript was normalised against the expression of the housekeeping gene porphobilinogen deaminase (PBGD). Standard curves were constructed with 10-fold serial dilutions of cDNA obtained from non-cancerous oesophageal mucosal cell layers of tissue samples from 10 cases as a standard mixture. The sequences of PCR primers for PBGD, MRP2 were as follows: forward primer 5′-TGTCTGGTAACGGCAATGCGGCTGCAAC-3′, reverse primer 5′-TCAATGTTGCCACCACACTGTCCGTCT-3′ used for amplification of PBGD, forward primer 5′-TAATGGTCCTAGACAACGGG-3′, reverse primer 5′-GGGCCTTCTGCTAGAATTT-3′ for MRP2. The PCR cycling condition was set as follows: an initial denaturing step at 95°C for 10 min and 40 cycles at 95°C for 15 s, 58°C for 10 s, and 72°C for 25 s. The relative amount of cDNA in each sample was measured by interpolation on the standard curve, and then the relative ratio of MRP2/PBGD mRNA expression in log2 scale was calculated for each ESCC sample.

### Knockdown analysis using MRP2-siRNAs

Two small-interfering RNA (siRNA-1, -2) of MRP2 (HSS102057, HSS174719) and negative control (NC) (Medium GC duplex of stealth RNAi NC duplexes) were purchased from Invitrogen. Among the eight ESCC cell lines supplied by RIKEN cell bank (Tsukuba, Japan), TE14 cells showed the highest MRP2 mRNA expression and were subsequently transfected with 15 nmol l^−1^ siRNA using Lipofectamine RNAiMAX (Invitrogen) in Opti-MEM I Reduced Serum Medium (Invitrogen). After 24 h, the medium was replaced by standard medium, and then 96 h from the siRNA administration, cells were collected for the following growth inhibitory assay as described below.

### Growth inhibitory assay

Cells (TE14, 1 × 10^4^ cells per well) were added in triplicate to a 96-well microplate, and after overnight incubation, the medium was replaced with 100 *μ*l of fresh medium containing various concentrations of DXR and CDDP, both of which chemoagents have been reported to be transported by MRP2 in some types of cell lines. The TE14 cells suspended in complete medium were used as a control for cell viability. After 4h (DXR and CDDP) treatment, the cells were washed with fresh medium. The number of viable cells was assessed by the 3-(4-, 5-dimethylthiazol-2-yl)-2, 5-dyphenyl tetrazolium bromide (MTT) (Sigma, St Louis, MO, USA) assay. Briefly, 10 *μ*l (50 *μ*g) of MTT were added to each well after 48 h (DXR and CDDP) from the chemoadministration. The plate was incubated for 4 h at 37°C, followed by removal of medium and the addition of 100 *μ*l of 2-propanol to each well to dissolve the resultant formazan crystals. Plate absorbance was measured in a microplate reader at a wavelength of 650 nm. After a pulsed exposure, the IC_50_ was calculated as percentage of control cultures that were not exposed to chemoagents using an interpolated logarithmic concentration curve. Results were derived from three independent sets of triplicate experiments.

### Statistical analysis

Data are expressed as mean±s.d. Correlations between MRP2 expression and various clinico-pathological parameters were each evaluated by the *χ*^2^ test and Fisher's exact probability test. Differences in continuous parameters between two groups were evaluated by the Mann–Whitney's *U*-test. Prognostic variables were assessed by log-lank test, and OS was analysed by Kaplan–Meier method. These analyses were carried out using SPSS for Windows v10 (SPSS, Chicago, IL, USA). A *P*-value of less than 0.05 denoted the presence of statistical significance.

## Results

### MRP2 protein expression by immunohistochemistry in ESCC and its correlation with clinico-pathological parameters

**Figure 1:**
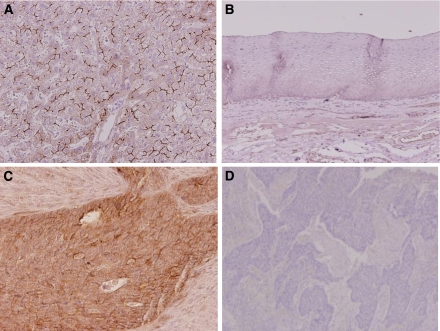
MRP2 expression by immunohistochemistry. (**A**) Strong MRP2 expression in liver tissue as a positive control (magnification, × 400). (**B**) Representative normal squamous epithelium negative for MRP2 expression (magnification, × 200). (**C**) Representative MRP2-positive ESCC showing staining mainly in the membrane and cytoplasm of tumour cells (magnification, × 200). (**D**) Representative MRP2-negative oesophageal squamous cell carcinoma with no appreciable staining of tumour cells (magnification, × 200).

A total of 81 samples that contained both cancerous and non-cancerous lesions were evaluated for MRP2 protein expression by IHC analysis. As a positive control, liver tissue showed strong MRP2 immunostaining mainly in the hepatocyte plasma membrane (Figure 1A). No normal squamous epithelium showed significant levels of immunostaining (Figure 1B). Of all samples, 14 (17.3%) showed positive MRP2 expression, mainly in the cell membrane and cytoplasm of tumour cells (Figure 1C), whereas the remaining 67 (82.7%) were negative for MRP2 expression (Figure 1D). The positive staining was almost homogeneous in single-cancer nests and among different areas (surface, central, and deepest areas) of the cancer lesion.

Table 1 lists the correlations between MRP2 expression and various clinico-pathological parameters. Of note, MRP2 expression was exceptional in the ESCC patients without NACT (2 out of 37, 5.4%), but was significantly more frequent in patients after NACT (12 out of 44, 27.3%). Women tended to have a higher rate of MRP2 expression than men (44.4 *vs* 13.9%, respectively), although the difference was small. Other clinico-pathological parameters including age, histological type, tumour location, pT, pN, and pStage were not associated with MRP2 expression.

**Figure 2:**
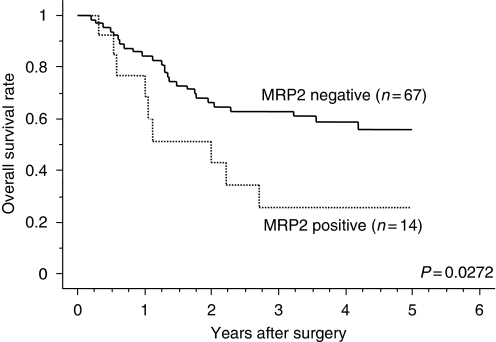
Survival curves according to MRP2 expression. Overall survival curve classified according to MRP2 expression for all patients were plotted by Kaplan–Meier method. Differences between two groups were evaluated by log–rank test. Ordinate: overall survival rate, abscissa: time after surgery (years).

Disease recurrence after curative resection was diagnosed in 35 (46.7%) of 75 patients with curative resection (R0) and the mean time to recurrence was 10.5 months. A total of 35 (43.2%) patients died and their average survival time from diagnosis to death was 1.4 years (range 0.2–4.2 years). The total 5-year OS rate was 50.9% and MRP2-positive patients showed a significantly poorer prognosis than MRP2-negative patients (5-year OS 55.7 *vs* 25.6%) (Figure 2).

### MRP2 mRNA expressions in resected specimens and endoscopy biopsy samples

**Figure 3:**
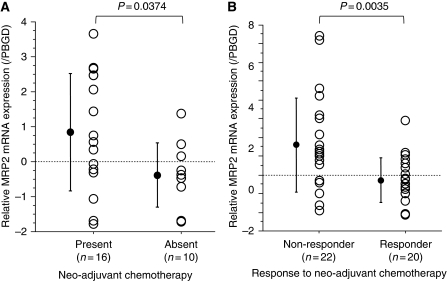
Differences in MRP2 mRNA expression between patients with and without neo-adjuvant chemotherapy in resected specimens (**A**), and between responders and non-responders at biopsy (**B**). (**A**) The relative ratio of MRP2 mRNA expression in resected tumours treated with neo-adjuvant chemotherapy (*n*=16) was significantly higher than in untreated cancers (*n*=10). (**B**) In endoscopy biopsy samples, the relative ratios of MRP2 mRNA expression in responders (*n*=22) were significantly higher than those in non-responders (*n*=20). Data are shown as mean±s.d. (log2 values).

RT–PCR analysis was performed to quantify the expression of MRP2 mRNA in surgically removed specimens from 26 representative cases, including 16 with NACT and 10 without NACT. MRP2 mRNA expression in tumours with NACT was 2.1-fold higher than in those without NACT, although there was no significant difference in TNM stage and other clinico-pathological parameters between the groups (data not shown) (Figure 3A).

The association between MRP2 mRNA expression and the effect of NACT was investigated in biopsy samples before NACT from 42 patients; the response of these patients to NACT was classified as non-responder in 22 and responder in 20. As shown in Figure 3B, MRP2 mRNA expression in non-responders was 2.9-fold higher than that in responders. Again, although these 42 samples were all advanced tumours with clinically positive lymph node metastases, there was no significant difference in clinical background parameters between the groups (data not shown).

### Association between MRP2 mRNA expression and chemoresistance in ESCC cancer lines

**Figure 4:**
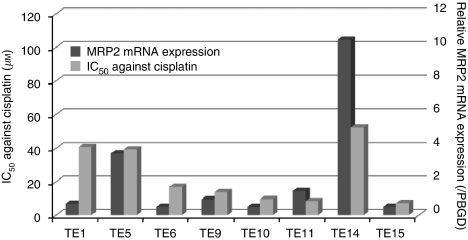
Correlation between MRP2 mRNA expression and CDDP-resistance (IC_50_) in eight cell lines of ESCC. Relatively high MRP2 expression was observed in TE14 and TE5 cell lines, both of which displayed strong resistance to CDDP. Black bar: the relative ratio of MRP2 mRNA expression, grey bar: IC_50_ values against CDDP.

To explore whether MRP2 expression functions specifically in chemoresistance to CDDP, we tested for a correlation between MRP2 mRNA expression and CDDP resistance (IC_50_) in eight ESCC cell lines (Figure 4). Relatively high MRP2 expression was observed in TE14 and TE5 cell lines, both of which displayed strong resistance to CDDP. Regression analysis showed a significant correlation between MRP2 mRNA expression and IC_50_ against CDDP (*R*=0.741, *R*2=0.549), suggesting that ESCC cell lines with higher MRP2 mRNA expression were more resistant to CDDP compared with those showing lower MRP2 expression.

To confirm these findings by an alternative approach, we transfected MPR2 siRNAs into the TE14 line, which had the highest cellular MRP2 expression. The specific gene silencing started 48 h after the administration of siRNA (two siRNAs for MRP2 with different sequences were used: siRNA-1 and siRNA-2) and continued for 144 h, which was examined by quantitative PCR, resulting in 63.8% (siRNA-1) and 65.9% (siRNA-2) of peak MRP2 downregulation compared with NCs. The knockdown effect was stable during this period. As shown in Table 2, downregulation of MRP2 conferred increased sensitivity to CDDP, but not to DXR. IC_50_ values against CDDP were significantly lower in TE14 cell lines transfected with siRNA-1 and siRNA-2 compared with those transfected with NC (20.5±1.4, 17.8±1.2 *vs* 32.4±1.2 *μ*M, (siRNA-1 *vs* NC); *P*=0.0003, (siRNA-2 *vs* NC); *P*=0.0005). On the other hand, IC_50_ values of DXR were almost similar among TE14 cells transfected with siRNA-1, siRNA-2, and NC (5.8±0.47, 5.4±0.54 *vs* 6.2±0.16 *μ*M, (siRNA-1 *vs* NC); *P*=0.2869, (siRNA-2 *vs* NC); *P*=0.2285).

## Discussion

To our knowledge, this study is the first to identify the clinical significance of MRP2 expression in chemoresistance in ESCC. Such a relationship was strongly suggested by the findings that (1) MRP2 expression in the clinical biopsy samples before NACT was significantly negatively correlated with the effect of NACT, and (2) in the cultured cell line, artificial MRP2 downregulation resulted in increased resistance to the chemotherapy. Furthermore, the clinical samples of patients treated with NACT showed significantly higher expression of MRP2 at both the protein and mRNA levels than those without NACT, and the increased MRP2 expression was associated with poor prognosis. Although complicated, these clinical observations implicated MRP2 in the acquired resistance to chemotherapy commonly encountered in ESCC patients.

Intrinsic or acquired drug resistance is a major factor limiting the effectiveness of chemotherapy in various cancers including ESCC. Drug resistance by tumours occurs not only to a single cytotoxic agent but also in the form of cross-resistance to many agents called MDR. One of the major mechanisms of MDR is an increased ability of tumour cells to actively efflux drugs, decreasing the intracellular drug accumulation. This mechanism is mediated by ATP-dependent drug efflux pumps known as ABC transporters (Leonard *et al*, 2003; Ozben, 2006). To date, at least 48 human ABC transporters have been identified, and they have been divided into seven sub-families, ABC-A through ABC-G. The first ABC transporter identified in this context was P-glycoprotein (PgP, MDR1, ABCB1) (Kartner *et al*, 1983), and in the absence of overexpressed MDR1, the protein MRP1, ABCC1 was discovered because of the MDR phenotype (Cole *et al*, 1992).

Cisplatin resistance is not a feature of MDR phenotypes conferred by either MDR1 or MRP1 (Borst *et al*, 2000). The finding that ABC transporter MRP2 could mediate active efflux of CDDP conjugated to glutathione (Taniguchi *et al*, 1996), supported by evidence that intracellular glutathione levels were related to CDDP toxicity (Ozols, 1985), suggested a possible role for active efflux as a resistance mechanism. In addition, human carcinoma cell line studies showed increased levels of MRP2 mRNA associated with relative CDDP resistance, decreased intracellular accumulation of CDDP, and decreased DNA adduct formation (Kool *et al*, 1997; Liedert *et al*, 2003). In ESCC cell lines (TE2, TE13) Tanaka *et al* (2010), in their analysis of the intracellular localisation of CDDP by using in-air micro-particle induced X-ray emission, recently reported that TE2 cells, which express lower MRP2 than TE13, had higher intracellular CDDP concentrations and sensitivity than TE13 cells. This is also in agreement with our present *in vitro* data regarding CDDP. In human tissue samples, accumulating evidence indicates that MRP2 expression is also associated with intrinsic CDDP resistance in the clinical setting, using tissues obtained from patients with colorectal cancer (Hinoshita *et al*, 2000), small-cell lung carcinoma (Ushijima *et al*, 2007), and ovarian cancer (Surowiak *et al*, 2006). These results are also consistent with our data from cancer tissue samples, although our *in vitro* data involving each single agent could not necessarily be translated directly to a clinical response to combination chemotherapy because of possible synergistic effects. However, in contrast with these data, other studies failed to show a significant association between MRP2 expression and chemosensitivity in patients with ovarian cancer (Arts *et al*, 1999; Materna *et al*, 2004) or lung cancer (Filipits *et al*, 2007; Kim *et al*, 2009). It therefore seems likely that multiple factors such as drug accumulation, DNA repair capacity, and apoptotic sensitivity contribute to clinical tumour chemosensitivity, and a mechanistic relationship could be difficult to detect amongst an unselected patient cohort in which a number of other factors also affect clinical outcome. As clinical significance of MRP2 other than chemosensitivity, Gan *et al* (2010) reported that MRP2 expression was significantly higher in poorly differentiated ESCC tumours compared with moderate or well differentiated ones, which was not observed in our study.

In terms of the contribution to acquired chemoresistance, the present IHC and qRT–PCR data showed higher MRP2 expression in resected tumours with NACT compared with those without NACT, implying residual tumours after NACT acquired the feature of chemoresistance. Unfortunately, we could not compare MRP2 expression levels in cancer tissues from the same patient before and after NACT because no samples were available. Nooter *et al* (1998) reported significantly higher MRP expression, although not specific MRP2 expression, in ESCC tumours from non-responders to CDDP-based chemotherapy when comparing MRP levels in paired tumour samples before and after chemotherapy, suggesting that chemotherapy was selected for drug-resistant cell clones. Furthermore, other *in vitro* analyses by Noma *et al* (2008) established two CDDP-resistant pancreatic cancer cell lines (SUIT-2-CD3 and SUIT-2-CD4) by continuously administering 10 nM CDDP for 3 and 4 months, respectively. Results of RT–PCR indicated that induction of MRP2 mRNA expression was significantly increased by 1.5- and 2.5-fold in SUIT-2-CD3 and SUIT-2-CD4 cells, respectively, compared with parent cells, whereas MRP1 and MRP3 expression remained unchanged, implying a contribution of MRP2 to acquired resistance for CDDP in pancreatic cancer.

An important observation regarding the functional significance of MRP2 expressed in tumour cells could be the sub-cellular localisation. In normal tissues, MRP2 is expressed in functionally polarised cells in which it specifically localises to the apical membrane of these cells. Apical localisation has also been described in tumours arising from these sites, a feature attributed to a targeting signal in the C-terminus of the MRP2 molecule (Harris *et al*, 2001). Single-nucleotide polymorphisms in MRP2 have been described that result in cytoplasmic localisation of the protein and that may reduce *in vivo* function (Hirouchi *et al*, 2004). Reduced CDDP sensitivity has also been reported in polarised mammalian kidney cells transfected with appropriately localised MRP2 (Cui *et al*, 1999). Furthermore, data of Surowiak *et al* (2006) indicated that MRP2 could confer resistance to CDDP in ovarian carcinoma only when expressed at the nuclear membrane, and this was supported by *in vitro* data (Materna *et al*, 2006). Although our IHC results showed MRP2-positive staining of both cytoplasm and membrane in tumour cells, MRP2 protein located in the cell cytoplasm might not function as an efflux pump (Evers *et al*, 1998). Further analysis focusing on the sub-cellular localisation of MRP2, and on the functional and clinical significance of such cellular location, is needed to elucidate the specific mechanism of chemoresistance induced by MRP2 in ESCC.

In conclusion, MRP2 expression seems to be associated with intrinsic resistance to chemotherapy in patients with ESCC, and is likely to also have a role in acquired chemoresistance. Further studies with larger cohorts are warranted to verify these results prospectively. The findings of this study open the door for exploration of efficacious treatment strategies and development of new therapeutic approaches for ESCC.

## Figures and Tables

**Table 1:** Correlation between MRP2 expression by immunohistochemistry and various clinico-pathological parameters

	**MRP2 expression**			
**Parameter**	**Positive**	**Negative**	**Total**	***P*-value**
*Age (years)*				
<65	8 (16.3)	41 (83.7)	49	0.7731
⩾65	6 (18.8)	26 (81.2)	32	
				
*Gender*				
Male	10 (13.9)	62 (86.1)	72	0.0435
Female	4 (44.4)	5 (55.6)	9	
				
*Histopathology*				
Well-, moderately differentiated	10 (16.7)	50 (83.3)	60	0.7500
Poorly differentiated	4 (19.0)	17 (81.0)	21	
				
*Location*				
Upper, middle thoracic oesophagus	6 (11.5)	46 (88.5)	52	0.1227
Lower thoracic oesophagus	8 (27.6)	21 (72.4)	29	
				
*Neo-adjuvant chemotherapy*				
Yes	12 (27.3)	32 (72.7)	44	0.0161
No	2 (5.4)	35 (94.6)	37	
				
*pT*				
T0–2	4 (16.7)	20 (83.3)	24	>0.9999
T3–4	10 (17.5)	47 (82.5)	57	
				
*Number of pN*				
<4	6 (11.1)	48 (88.9)	54	0.0594
⩾4	8 (29.6)	19 (70.4)	27	
				
*pStage*				
Stages 0–2	4 (12.1)	29 (87.9)	33	0.3800
Stages 3–4	10 (20.8)	38 (79.2)	48	

pT, pN, pStage (pathological classification) according to TNM classification.

**Table 2:** Modulation of resistance against cisplatin and doxorubicin by MRP2 siRNA

	**IC_50_**	
	**Cisplatin (*μ*M)**	**Doxorubicin (*μ*M)**
TE14 NC	32.4 (±1.2)	6.2 (±0.16)
TE14 siRNA-1	20.5 (±1.4)^a^	5.8 (±0.47)^b^
TE14 siRNA-2	17.8 (±1.2)^c^	5.4 (±0.54)^d^

Abbreviations: NC=negative control; IC_50_=half maximal inhibitory concentration; siRNA=small-interfering RNA.

a *P*=0.0003, compared with NC.

b *P*=0.2869, compared with NC.

c *P*=0.0005, compared with NC.

d *P*=0.2285, compared with NC.

Data are shown as mean±s.d.
